# Novel High Holding Voltage SCR with Embedded Carrier Recombination Structure for Latch-up Immune and Robust ESD Protection

**DOI:** 10.1186/s11671-019-3017-8

**Published:** 2019-05-28

**Authors:** Zhuo Wang, Zhao Qi, Longfei Liang, Ming Qiao, Zhaoji Li, Bo Zhang

**Affiliations:** State Key Laboratory of Electronic Thin Films and Integrated Devices, University of Electronic Science and Technology of China, Chengdu, 610054 Sichuan China

**Keywords:** Electrostatic discharge (ESD), Silicon-controlled rectifier (SCR), Holding voltage (*V*_h_), Latch-up, Transmission line pulse (TLP)

## Abstract

A novel CMOS-process-compatible high-holding voltage silicon-controlled rectifier (HHV-SCR) for electrostatic discharge (ESD) protection is proposed and demonstrated by simulation and transmission line pulse (TLP) testing. The newly introduced hole (or electron) recombination region H-RR (or E-RR) not only recombines the minority carrier in parasitic PNP (or NPN) transistor base by N+ (or P+) layer, but provides the additional recombination to eliminate the surface avalanche carriers by newly added P+ (or N+) layer in H-RR (or E-RR), which brings about a further improvement of holding voltage (*V*_h_). Compared with the measured *V*_h_ of 1.8 V of low-voltage triggered silicon-controlled rectifier (LVTSCR), the *V*_h_ of HHV-SCR can be increased to 8.1 V while maintaining a sufficiently high failure current (*I*_t2_ > 2.6 A). An improvement of over four times in the figure of merit (FOM) is achieved.

## Introduction

With the development of semiconductor integrated technology and the consistent miniaturization of semiconductor device’s feature size, the device damage induced by ESD is becoming more severe. At the cost of large chip area, the conventional devices such as diode and gate grounded N-channel MOSFET (ggNMOS) featuring normal ESD robustness were reported [1]. In order to realize improved ESD capability with a smaller device dimension, the low-voltage triggered silicon-controlled rectifier (LVTSCR) has been considered as an attractive device due to its high-current capability per unit area [2]. For low-voltage applications, owing to the embedded low-trigger voltage (*V*_t1_) ggNMOS, the LVTSCR with excellent ESD robustness is capable of providing faster ESD response speed than that obtained in conventional SCR. However, the strong inherent positive feedback causes an extremely low *V*_h_ (1~2 V), which is responsible for latch-up and transient mis-trigger [3]. Such negative effects can be effectively suppressed by simply increasing *V*_h_ [3–11]. The device will be free from the latch-up and transient mis-trigger, while the *V*_h_ is higher than the power supply voltage (VDD). Accordingly, The N+ESD region and P+LDD region were added into SCR with additional masks and ion implant steps to improve *V*_h_ [3]. However, the ESD robustness may deteriorate due to the additional power dissipation together with the increased *V*_h_. In addition, the emitter voltage clamp technology for *V*_h_ improvement with acceptable failure current (*I*_t2_) was also proposed [5]. Nevertheless, the *V*_h_ in the aforementioned approaches is non-adjustable which still presents inconvenience and limitation in versatile applications.

In this letter, a novel high-holding voltage silicon-controlled rectifier (HHV-SCR) is proposed and demonstrated by TCAD simulation and TLP testing. The device simultaneously achieves high *V*_h_, high *I*_t2_, and adjustable *V*_h_ without any additional masks and steps. The TLP-test was carried out to validate that the *V*_h_ can be effectively improved while maintaining a sufficiently high *I*_t2_. According to the tested results, the HHV-SCR features over four times higher *V*_h_ than that in the LVTSCR with the negligible degradation in *I*_t2_.

## Method

In this work, a novel high-holding voltage SCR with an embedded carrier recombination structure is investigated. The physical models IMPACT.I, BGN, CONMOB, FLDMOB, SRH, and SRFMOB are used in numerical simulation. Based on the model, H-RR and E-RR are optimized to achieve high *V*_h_ and high *P*_M_. The fabricated HHV-SCRs and LVTSCR are tested by TLP system.

## Structure and Mechanism

**Fig. 1:**
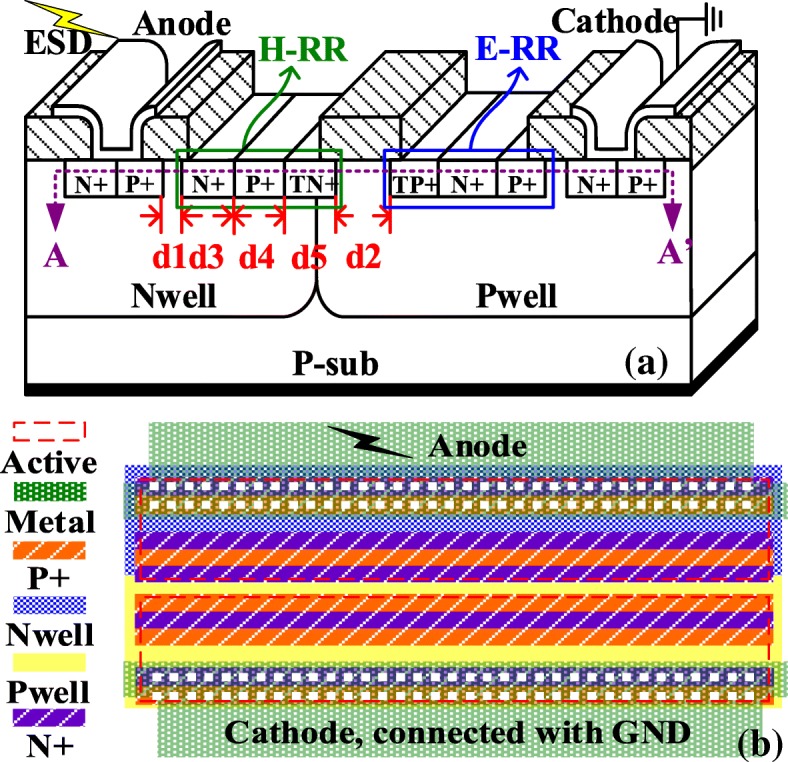
**a** The schematic cross-sectional view of proposed HHV-SCR. **b** The layout diagram of proposed HHV-SCR

The schematic cross-sectional view of the proposed HHV-SCR and layout diagram are shown in Fig. 1a, b, respectively. The newly introduced H-RR and E-RR formed by floating N+ and P+ are identical to the N+ and P+ in the anode and cathode areas, respectively. The floating N+ in H-RR (or floating P+ in E-RR) is placed next to the P+ region in the anode (or N+ region in the cathode). Moreover, the new floating P+ in H-RR (or floating N+ in E-RR) is also located next to the aforementioned floating N+ in H-RR (or floating P+ in E-RR). The low-trigger N+ in H-RR (TN+) and low-trigger P+ in E-RR (TP+) are also fabricated by the same processes as the N+ (or P+) region in the anode (or cathode) to ensure the *V*_t1_ within an acceptable range. As a positive ESD voltage (*V*_ESD_) rising up to a certain level, the TN+/P-well/TP+ junction with a low-breakdown voltage will breakdown first followed by the snapback of the parasitic transistors triggered by the avalanche current. The strong positive feedback of the parasitic BJTs is responsible for the considerably low *V*_h_ of the LVTSCR. In the HHV-SCR, the N+ in H-RR (or the P+ in E-RR) will recombine the minority carriers injected from the edge of anode P+ (or cathode N+), which reduces the current gain (*β*) of the parasitic PNP (or NPN) and eliminates the surface bipolar effect. Importantly, the P+ in H-RR (or the N+ in E-RR) blocks the surface low-resistance path by recombining the surface electrons (or holes). Compared with the H-RR without P+ (or E-RR without N+), the new P+ in H-RR (or the N+ in E-RR) provides the additional recombination to eliminate the surface electrons (or holes) injected from cathode (or anode) and those induced by impact ionization (shown in Fig. 3a), which brings about the further increasing of *V*_h_. By combining these modifications, a significant improvement in *FOM* is verified. The figure of merit (FOM) is cited from [7] and defined as the tolerable power density of single device given by FOM=(*V*_h_·*I*_t2_)/(*N*·*W*) to evaluate the *V*_h_ and *I*_t2_ performance of single device. Generally, accompanied by the improving of *V*_h_ performance, it still causes the degradation of *I*_t2_ due to the higher-power dissipation. Therefore, the higher FOM signifies the single device can achieve the higher current capability on the higher *V*_h_ level (*N* is the number of the stacking device; *W* is the device width).

## Results and Discussion

### Simulated results

**Fig. 2:**
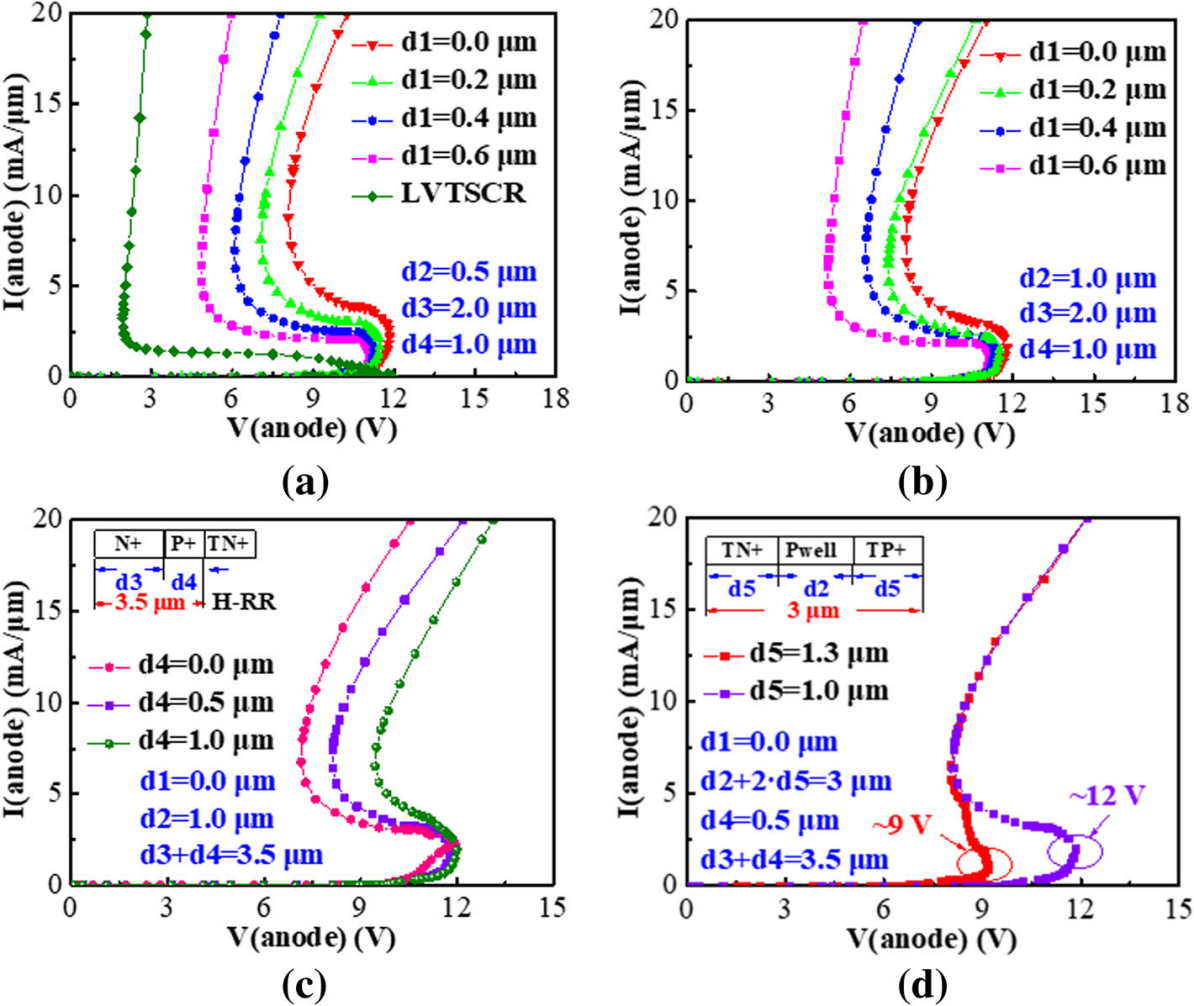
Simulated snapback I-V characteristics of conventional LVTSCR and proposed HHV-SCR with the d1 increasing from 0 μm to 0.6 μm at **a** d2 = 0.5μm and **b** d2 = 1μm. **c** The I-V curves of HHV-SCR with different d3 and d4 for the fixed d3 + d4 (d3 + d4 = 3.5 μm). **d** The I-V curves of HHV-SCR with various *V*_t1_

**Fig. 3:**
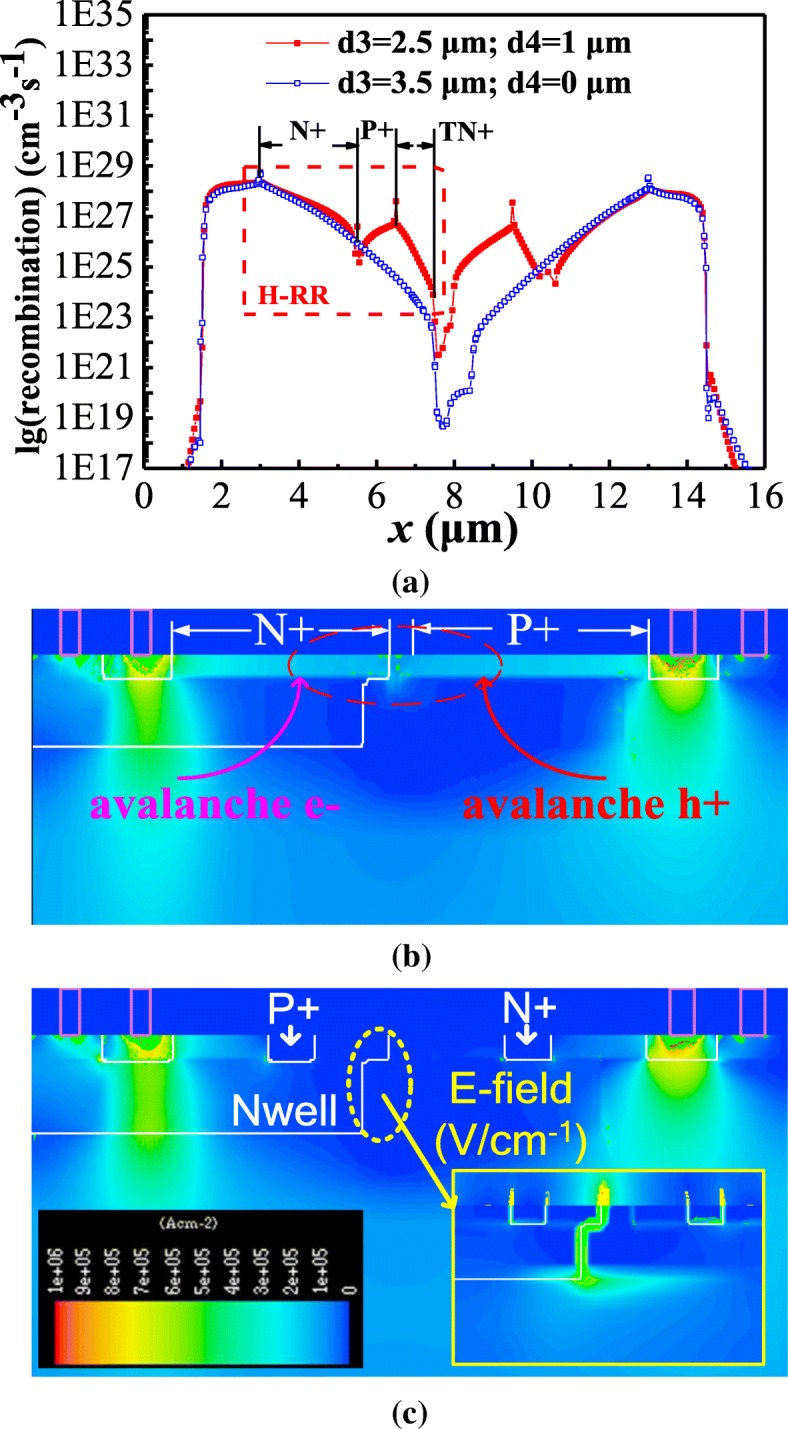
**a** The recombination distribution curves, and the current distributions of HHV-SCR with (**b**) d3 = 3.5 μm, d4 = 0 μm, and (**c**) d3 = 2.5 μm, d4 = 1 μm

The device characteristics were studied and simulated by TCAD Medici, where the corresponding models such as impact ionization and concentration-dependent mobility model were used. The simulated I-V curves of the LVTSCR and HHV-SCRs are shown in Fig. 2. The *V*_h_ of the LVTSCR is as low as 1.8 V, while the *V*_h_ of the HHV-SCR is improved from 4.6 V to 8.1 V with d1 decreased from 0.6 μm to 0 μm for d2 = 0.5 μm. In fact, the smaller d1 is favored for improved recombination capability of N+ in H-RR (or P+ in E-RR) to obtain a lower *β*, which explains that the HHV-SCR always achieves the highest *V*_h_ for d1 = 0 μm. The simulated results in Fig. 2b indicate that the *V*_h_ of HHV-SCR is further improved with d2 increased from 0.5 to 1 μm due to the increasing of device length. For demonstration, the P+ in H-RR (or N+ in E-RR) is also a key factor to increase *V*_h_. The simulated results are shown in Fig. 2c. When the H-RR (or E-RR) with fixed d3 + d4 is completely formed by heavy doping N+ (or P+) (e.g., d3 = 3.5 μm, d4 = 0 μm), the simulated *V*_h_ is 7.1 V. By inserting the P+ inside H-RR and N+ inside E-RR with fixed d3 + d4 (e.g., d3 = 2.5 μm, d4 = 1.0 μm), the simulated *V*_h_ can be increased up to about 9.5 V. It can be inferred that the new P+ in H-RR (or N+ in H-RR) is effective in recombining surface avalanche electrons (or holes) to block the surface current path. Therefore, a higher *V*_h_ is required for the HHV-SCR to sustain the same holding current (*I*_h_). The recombination curve alone AA′ line shown in Fig. 3a demonstrates the increasing of recombination rate induced by new P+ in H-RR (or N+ in E-RR). The TN+ and TP+ are adopted to ensure the *V*_t1_ within an acceptable range. By adjusting the d2 and d5 at the fixed d5 + d2 + d5, the *V*_t1_ of HHV-SCR can be significantly reduced from 12 V to 9.0 V to meet the design window of 5 V circuits with the negligible impact on *V*_h_, shown in Fig. 2d. The current distribution diagrams of the simulated devices at the holding point are also shown in Fig. 3b, c, respectively. Compared with the current distribution in the HHV-SCR with d3 = 3.5 μm, d4 = 0 μm, the surface current path in proposed HHV-SCR is blocked due to the additional recombination rate benefited from P+ in H-RR and the N+ in E-RR.

### Experimental Results

**Fig. 4:**
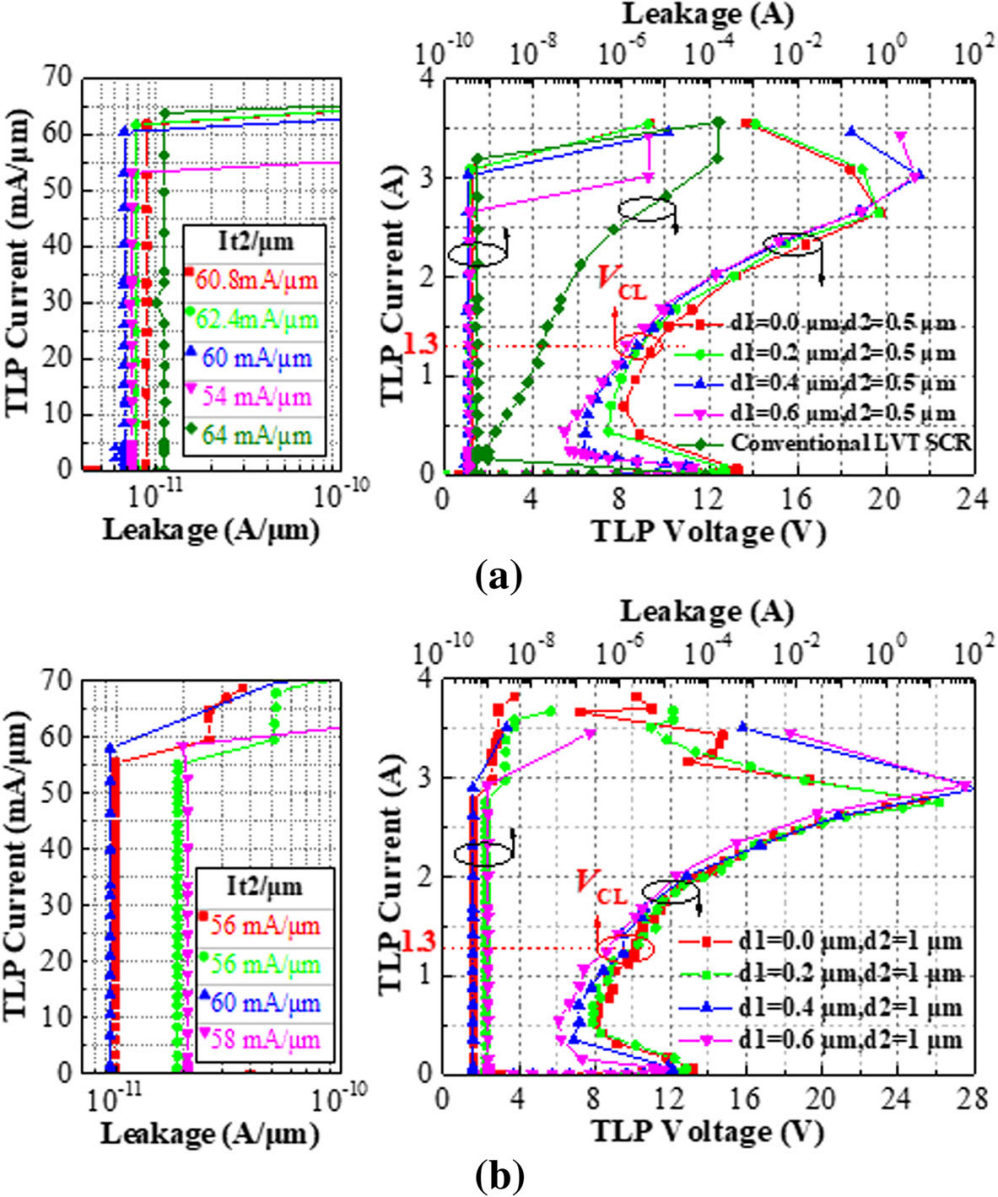
Experimental failure current at the unit width and corresponding TLP I-V characteristics of conventional LVTSCR and proposed HHV-SCRs with **a** d2 = 0.5 μm and **b** d2 = 1 μm at *W* = 50 μm

**Fig. 5:**
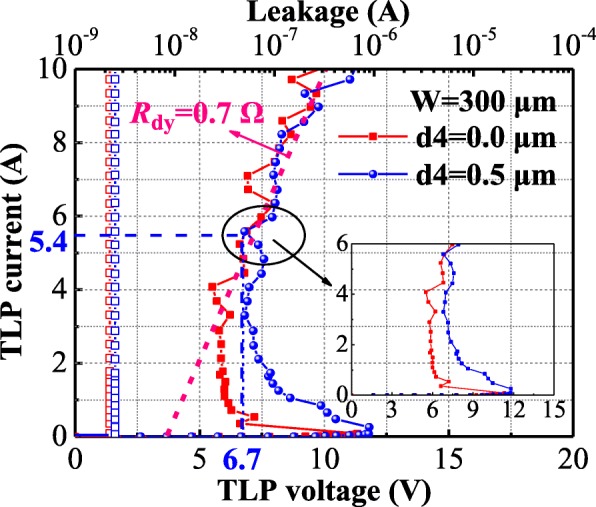
Experimental TLP characteristic of HHV-SCR with d4 = 0.0 μm and d4 = 1.0 μm at d1 = 0.6 μm, *W* = 300 μm

The fabricated devices are tested by TLP system. The total widths (W) of all tested SCR are 50 μm and with single finger for the parameter’s comparison (Table 1). All of the tested devices occupy the similar layout area. The device parameters are shown in Table 2. Figure 4a shows the TLP measurement curves of the HHV-SCRs with d2 =0.5 μm (called devices B1) and the LVTSCR. According to the experimental results, the *V*_h_ of HHV-SCR is increased from 5.5 to 8.0 V with the d1 decreased from 0.6 μm to 0.0 μm, which is much higher than 1.8 V obtained in the conventional LVTSCR. As the d2 increases from 0.5 to 1 μm, the corresponding HHV-SCRs (called devices B2) obtain a higher *V*_h_ shown in Fig. 4b. Considering the design window, the clamping voltage (*V*_CL_) under the given index is also a key parameter to evaluate clamping ability. From the tested results, the *V*_CL_ of single finger HHV-SCR is also kept within the acceptable range at the HBM = 2 kV (*I*_TLP_=1.3 A) although the finger width is only 50 μm. However, all devices cannot provide the eligible *V*_CL_ under the stronger ESD stress due to the high *V*_h_ and large dynamic resistance (*R*_dy_) induced by undersized device width. For satisfying the higher on-chip ESD requirement, the finger width is extended to the acceptable 300 μm for d1 = 0.6 μm, d4 = 0.5 μm, and d1 = 0.6 μm, d4 = 0 μm. The TLP testing shown in Fig. 5 demonstrates that the HHV-SCR with d4 = 0.5 μm features the extremely low *R*_dy_ (about 0.7 Ω), superior ESD robustness (*I*_t2_ > 10 A) and high *V*_h_ of 6.7 V. It can be observed that the *V*_CL_ is as low as 6.7 V at the *I*_TLP_ = 5.4 A (HBM = 8 KV). Furthermore, the higher *V*_h_ benefited from P+ in H-RR (or N+ in E-RR) is also proved, as compared with the TLP curve of SCR with d4 = 0 μm. The tested results of 50 μm single-finger devices are listed in Table 1.

**Table 1:** Comparison of experimental results

Experimental devices			L/W (μm/μm)	*V*_h_ (V)	*I*_t2_ (A)	*V*_CL *@1.3*__A_ (V)	FOM (mW/μm)
Conventional LVTSCR			15/50	1.8(< 5)	3.2	4.8	110
Proposed HHV-SCR	d2 (μm)	d1 (μm)	L/W (μm/μm)	*V*_h_ (V)	*I*_t2_ (A)	*V*_CL *@1.3*__A_ (V)	FOM (mW/μm)
	0.5	0.0	17/50	8.0	3.04	8.5	490
		0.2	17.4/50	7.4	3.12	8.9	460
		0.4	17.8/50	6.1	3.0	8.9	370
		0.6	18.2/50	5.5	2.7	9.5	300
	1.0	0.0	17.5/50	8.1	2.8	8.4	450
		0.2	17.9/50	8.0	2.8	8.8	450
		0.4	18.3/50	7.0	3.0	8.8	420
		0.6	18.7/50	6.0	2.9	9.8	350

**Table 2:** List of Abbreviations

Abbreviation	Full name
HHV-SCR	High-holding voltage silicon-controlled rectifier
ESD	Electrostatic discharge
TLP	Transmission line pulse
H-RR	Hole recombination regions
E-RR	Electron recombination regions
ggNMOS	Gate grounded N-channel MOSFET
LVTSCR	Low-voltage triggered SCR

## Conclusion

A novel CMOS-process-compatibleHHV-SCR is studied and measured by TCAD simulation and TLP system. Compared with the conventional LVTSCR, the HHV-SCR features significantly improved *V*_h_ (an improvement of over 450% in the *V*_h_ is achieved) and without sacrificing the chip area. Furthermore, the *V*_h_ of the HHV-SCR can be adjusted from 5.5 V to 8.1 V to satisfy the different *V*_h_ requirements with negligible degradation in *I*_t2_. In terms of *P*_M_, compared with the conventional LVTSCR, over 200% improvement is also achieved.

## Data Availability

All data generated or analyzed during this study are included in this published article.
